# Appropriate body mass index cutoffs for type 2 diabetes in Xinjiang population: defining the influence of liver aminotransferase

**DOI:** 10.18632/oncotarget.28009

**Published:** 2021-07-06

**Authors:** Jing-Yuan Xu, Long-Bao Yang, Zhi-Yi Han, Kai Wang, Zhen-Hua Yin, Ting Wu, Yong Shao, Xiao-Lan Lu

**Affiliations:** ^1^Department of Gastroenterology, The Second Affiliated Hospital of Xi’an Jiaotong University, Xi’an 710004, China; ^2^Department of Gastroenterology, Shanghai Pudong Hospital of Fudan University, Shanghai 201399, China; ^3^Karamay Central Hospital of Xinjiang, Karamay 834099, China; ^4^Community Health Service Center of Jinxi Town, Kunshan 215300, China; ^*^These authors contributed equally to this work

**Keywords:** type 2 diabetes, alanine aminotransferase, aspartate aminotransferase, body mass index

## Abstract

Background/Purpose: Recent study suggested that type 2 diabetes (T2DM) attributed to body mass index (BMI) could be influenced by liver aminotransferase. We aim to ascertain the cut-off point of BMI associated with T2DM and the influence of both elevated aminotransferase (AST) and alanine aminotransferase (ALT).

Materials and Methods: In our retrospective cohort study, T2DM was diagnosed when FBS ≥ 7.0 mmol/L, BMI of participants with baseline fasting (FBS) < 7.0 mmol/L was divided by percentiles and by aminotransferanse (ALT and AST ≥ 20 U/L, ALT or AST < 20 U/L). Hazard ratios and the turning point of BMI of high T2DM risk was estimated in totality and different aminotransferanse groups.

Results: During an average follow-up time of 3.71 years of 33346 participants, 1486 developed T2DM, and the average baseline BMI of participants who developed T2DM was 26.22 kg/m^2^. Cumulative incidence of T2DM was more than 5% when ALT and AST ≥ 20U/L, age over 44, male sex or BMI over 25.39 kg/m^2^; The risk of T2DM incidence increased as the BMI grow. The turning point of BMI at high risk of T2DM was 25.0 kg/m^2^ in totality, 25.1 kg/m^2^ when ALT or AST < 20 U/L and 26.1 kg/m^2^ when ALT and AST ≥ 20U/L.

Conclusions: BMI of 25.0 kg/m^2^ was the cutoff point for T2DM development, and there is greater association between BMI and T2DM when ALT or AST < 20 U/L.

## INTRODUCTION

Type 2 diabetes (T2DM) is one of the most serious public health issues in the 21^st^ century and is becoming increasingly prevalent worldwide [1]. T2DM imposes a public health burden of mortality and disability, not only because of T2DM itself, but also its increased incidence and mortality from cancers, cardiovascular events and other diseases [1–3]. In China, the overall prevalence of T2DM in adults was 10.9% according to a study in 2013 [4], and the personal annual direct cost attributable to T2DM without complication, with 1, 2 and 3+complication was US$ 248, US$ 1399, US$ 1705 and US$ 2994, respectively [5].

Body mass index (BMI) is the most important risk factor of T2DM [6–9]. New lifestyle brought by urbanization and globalization such as fast food and sedentary office life has caused a tremendous overweight and obesity population, which significantly increases the incidence of T2DM [7, 9–11]. According to the national survey of obesity and metabolic syndrome, the average BMI in T2DM patients is 25.0 kg/m^2^, the prevalence of T2DM in BMI from 25.0 kg/m^2^ to 27.4 kg/m^2^ and above 27.4 kg/m^2^ is 12.8% and 18.5%, the prevalence in male adult is 33.7% and 13.7%, and that in female adult is 29.2% and 10.7%, respectively [8]. The BMI threshold associated with increased T2DM is modified by sex and ethnicity [7, 12, 13]. Recommended BMI cut-off point for preventing T2DM in different Asian populations were not consistent. A study based on Thai population derived the BMI cut-point to define T2DM risk by odds ratio as 22 [7]; Another multi-center research found BMI of 25 kg/m^2^ in Chinese is equivalent of BMI of 24 kg/m^2^ in South Asian for incidence rate of diabetes [12]. For this reason, it is important to ascertain the high risk BMI range in different populations to make strategy for controlling T2DM. Xinjiang autonomous region of China (Central Asian in geography) composed of 55 ethnic groups, with a prevalence of T2DM approximately 6.1% in rural areas 8.2% in urban areas [14]. It is meaningful in disease prevention to estimate the BMI threshold for increased T2DM based on adults in Xinjiang.

Liver dysfunction is also associated with T2DM, and the association between serum alanine aminotransferase (ALT), aspartate aminotransferase (AST) and T2DM was broadly replicated [15–17]. Recent studies suggested that the upper cut-off values may be lower than 40 U/L [18–21]. A study based on Korean showed the upper limit of normal values of AST were 25.35 U/L and 24.25 U/L for healthy men and women, respectively; and that of ALT were 22.15 U/L and 22.40 U/L for healthy men and women, respectively [20]. Also, a study of 6835 blood donors suggested that the upper limit of aminotransferase values was 31 U/L for men and 19 U/L for women [19]. Many studies reported the association between BMI and liver aminotransferase, and it is modified by sex and age [22–24]. Another study further indicated that T2DM risk attributed to BMI could be influenced by liver aminotransferase: in group of high levels of both AST and ALT and in group of high level of either AST or ALT, high BMI was independently associated with diabetes incidence, and in group of high levels of both AST and ALT, odds ratio was relatively higher than that in group of high levels of either AST and ALT; While in group of low levels of both AST and ALT, high BMI was not an independent risk factor for diabetes [25].

In this study, we aimed to find the cut-off point of BMI associated with high T2DM risk in Xinjiang population; we also aimed to find the difference of cut-off point brought by elevated liver aminotransferase.

## RESULTS

During an average follow-up time of 3.71 years of 33346 participants, 1486 developed T2DM, and the incidence density was 1.2/100 person-years. The average baseline BMI of people who developed T2DM was 26.22 (standard deviation (SD) 3.67) kg/m^2^ and that of people who did not develop T2DM was 24.18 (SD 3.50). After adjusting age, sex and BMI, AST ≥ 20 U/L and ALT ≥ 20 U/L showed statistical difference on T2DM incidence (HR 1.118 *P* = 0.037), while AST ≥ 20 U/L or ALT ≥ 20 U/L showed no statistical difference (HR 1.113 *P* = 0.109). For this reason, we define aminotransferase group 1 if AST ≥ 20 U/L and ALT ≥ 20 U/L, and group 2 if AST < 20 U/L or ALT < 20 U/L. Association between Baseline data and T2DM Incidence were shown in Table 1. The T2DM incidence was more than 5% for Aminotransferase group 1; In both aminotransferase group 1 and group 2, T2DM incidence was more than 5% for age over 44 years, male sex and BMI over 25.39 kg/m^2^.

**Table 1:** Association between baseline data and T2DM incidence and by aminotransferase

	Total			AST or ALT < 20			AST and ALT ≥ 20		
	T2DM incidence	%	*P*	T2DM incidence	%	*P*	T2DM incidence	%	*P*
							incidence		
**Total**	1486/33346	4.46		701/19383	3.62		785/13963	5.62	
**Age *years***									
Under 30	85/5734	1.48	<0.001	30/3702	0.81		55/2032	2.71	<0.001
30~44	461/14969	3.08		216/9063	2.38		245/5906	4.15	
45~59	663/9644	6.87		299/5007	5.97		364/4637	7.85	
60 or over	277/2999	9.24		156/1611	9.68		121/1388	8.72	
**Sex**									
Male	1086/18990	5.72	<0.001	477/9137	5.22		609/9853	6.18	<0.001
Female	400/14356	2.79		224/10246	2.19		176/4110	4.28	
**BMI *kg/m^2^***									
<19.05	18/1620	1.11	<0.001	14/1296	1.08	<0.001	4/324	1.23	<0.001
19.05~<19.96	21/1741	1.21		14/1314	1.07		7/427	1.64	
19.96~<20.66	36/1628	2.21		24/1179	2.04		12/449	2.67	
20.66~<21.22	35/1653	2.12		23/1193	1.93		12/460	2.61	
21.22~<21.72	42/1772	2.37		27/1239	2.18		15/533	2.81	
21.72~<22.22	50/1566	3.19		27/1064	2.54		23/502	4.58	
22.22~<22.67	44/1699	2.64		28/1163	2.41		16/536	2.99	
22.67~<23.14	46/1591	2.89		24/976	2.46		22/615	3.58	
23.14~<23.56	55/1750	3.14		28/1068	2.62		27/682	3.96	
23.56~<24.03	55/1590	3.46		26/942	2.76		29/648	4.48	
24.03~<24.46	76/1733	4.39		38/1000	3.8		38/733	5.18	
24.46~<24.91	77/1629	4.73		34/883	3.85		43/746	5.76	
24.91~<25.39	74/1690	4.38		42/906	4.64		32/784	4.08	
25.39~<25.91	89/1652	5.39		49/852	5.75		40/800	5	
25.92~<26.45	104/1744	5.96		46/807	5.7		58/937	6.19	
26.45~<27.10	96/1578	6.08		45/730	6.16		51/848	6.01	
27.10~<27.78	103/1709	6.03		37/732	5.05		66/977	6.76	
27.78~<28.89	145/1674	8.66		49/737	6.65		96/937	10.25	
28.89~<30.45	145/1673	8.67		54/659	8.19		91/1014	8.97	
≥30.45	175/1654	10.58		72/643	11.2		103/1011	10.19	
**Aminotransferase**									
Group 1^*^	785/13963	5.62	<0.001	NA	NA		NA	NA	
Group 2^**^	701/19383	3.62		NA	NA		NA	NA	

^*^Group 1: AST ≥ 20 U/L and ALT ≥ 20 U/L; ^**^Group 2: AST < 20 U/L or ALT < 20U/L; NA: not applicable.

The risk of T2DM incidence increased as the BMI grow. HR in each BMI group was shown in Table 2. The risk of T2DM incidence in BMI group of 24.03~<24.46 kg/m^2^ was 2.022 times statistically higher than that in the reference BMI group of 19.96~<20.66 kg/m^2^. After adjustment for sex, the risk of T2DM incidence in BMI group of 24.46~<24.91 kg/m^2^ was 1.796 times statistically higher than that in the reference BMI group. After adjustment for sex and age, the risk of T2DM incidence in BMI group of 24.91~<25.39 kg/m^2^ was 1.901 times statistically higher than that in the reference BMI group.

**Table 2:** Hazard ratio for T2DM incidence of BMI in overall cohort population

	Hazard Ratio (95% CI)		
	Model 1^a^	Model 2^b^	>Model 3^c^
**BMI *kg/m^2^***			
<19.05	0.543 (0.308,0.955)^*^	0.550 (0.312,0.968)^*^	0.601 (0.341,1.059)
19.05~<19.96	0.539 (0.315,0.924)^*^	0.553 (0.323,0.947)^*^	0.583 (0.340,0.999)
19.96~<20.66	1	1	1
20.66~<21.22	0.948 (0.595,1.510	0.937 (0.588,1.493)	0.920 (0.577,1.466)
21.22~<21.72	1.038 (0.665,1.620	1.009 (0.646,1.575)	0.935 (0.599,1.461)
21.72~<22.22	1.476 (0.961,2.266)	1.371 (0.891,2.109)	1.238 (0.804,1.908)
22.22~<22.67	1.189 (0.765,1.847)	1.085 (0.697,1.689)	0.965 (0.619,1.504)
22.67~<23.14	1.298 (0.839,2.007)	1.076 (0.689,1.681)	0.998 (0.640,1.556)
23.14~<23.56	1.428 (0.938,2.174)	1.214 (0.790,1.868)	1.069 (0.693,1.647)
23.56~<24.03	1.598 (1.050,2.433)	1.316 (0.850,2.040)	1.123 (0.724,1.743)
24.03~<24.46	2.022 (1.360,3.007)^**^	1.688 (1.113,2.561)	1.490 (0.982,2.262)
24.46~<24.91	2.299 (1.546,3.417)^***^	1.796 (1.179,2.737)^**^	1.606 (1.053,2.451)
24.91~<25.39	2.184 (1.465,3.256)^***^	1.787 (1.158,2.757)^**^	1.395 (0.909,2.139)
25.39~<25.91	2.644 (1.794,3.896)^***^	2.295 (1.516,3.473)^***^	1.901 (1.259,2.871)^**^
25.92~<26.45	2.847 (1.948,4.162)^***^	2.425 (1.593,3.691)^***^	1.970 (1.299,2.989)^**^
26.45~<27.10	3.032 (2.063,4.456)^***^	2.694 (1.763,4.117)^***^	2.222 (1.454,3.396)^***^
27.10~<27.78	2.989 (2.042,4.375)^***^	2.319 (1.524,3.530)^***^	1.940 (1.280,2.939)^**^
27.78~<28.89	4.23 (2.934,6.098)^***^	3.752 (2.535,5.552)^***^	3.080 (2.078,4.565)^***^
28.89~<30.45	4.559 (3.159,6.578)^***^	3.654 (2.441,5.471)^***^	3.111 (2.081,4.650)^***^
≥30.45	5.618 (3.918,8.056)^***^	5.202 (3.538,7.650)^***^	4.668 (3.164,6.887)^***^

^a^Crude HR; ^b^Sex adjusted; ^c^Sex and age adjusted; ^*^ *P* < 0.05; ^**^ *P* < 0.01; ^***^ *P* < 0.001.

Tables 3 and 4 showed the HR in each BMI group in group1 and group 2, separately. BMI of 24.03~<24.46 kg/m^2^ was associated with statistically higher T2DM incidence in both aminotransferase group 1 and group 2. After adjustment for sex, BMI of 24.03~<24.46 kg/m^2^ began to be associated with statistically higher T2DM incidence in group 1, and of 24.91~<25.39 kg/m^2^ in group 2. After adjustment for sex and age, BMI of 24.46~<24.91 kg/m^2^ and 25.39~<25.91 kg/m^2^ began to be associated with statistically higher T2DM incidence in group 1 and group 2, separately.

**Table 3:** Hazard ratio for T2DM incidence of BMI in aminotransferase group 1

	Hazard Ratio (95% CI)		
	Model 1^a^	Model 2^b^	Model 3^c^
**BMI *kg/m^2^***			
<19.05	0.442 (0.143,1.373)	0.442 (0.142,1.371)	0.475 (0.153,1.475)
19.05~<19.96	0.580 (0.228,1.474)	0.585 (0.230,1.488)	0.618 (0.243,1.574)
19.96~<20.66	1	1	1
20.66~<21.22	0.982 (0.441,2.187)	0.983 (0.442,2.189)	0.988 (0.444,2.200)
21.22~<21.72	1.039 (0.486,2.220)	1.029 (0.481,2.200)	0.936 (0.436,2.006)
21.72~<22.22	1.776 (0.883,3.570)	1.755 (0.867,3.554)	1.696 (0.837,3.437)
22.22~<22.67	1.164 (0.550,2.463)	1.104 (0.518,2.351)	1.057 (0.497,2.249)
22.67~<23.14	1.382 (0.684,2.794)	1.258 (0.613,2.581)	1.184 (0.578,2.425)
23.14~<23.56	1.502 (0.761,2.965)	1.364 (0.677,2.746)	1.271 (0.629,2.570)
23.56~<24.03	1.791 (0.914,3.512)	1.666 (0.831,3.343)	1.504 (0.748,3.025)
24.03~<24.46	2.031 (1.061,3.888)^*^	1.996 (1.012,3.937)^*^	1.875 (0.950,3.700)
24.46~<24.91	2.567 (1.350,4.882)^**^	2.361 (1.195,4.666)^*^	2.219 (1.119,4.397)^*^
24.91~<25.39	1.694 (0.872,3.292)	1.639 (0.796,3.375)	1.439 (0.701,2.954)
25.39~<25.91	2.117 (1.110,4.038)^*^	2.250 (1.133,4.468)^*^	2.007 (1.016,3.963)^*^
25.92~<26.45	2.410 (1.294,4.490)^**^	2.512 (1.288,4.899)^**^	2.235 (1.150,4.343)^*^
26.45~<27.10	2.530 (1.347,4.751)^**^	2.491 (1.266,4.900)^**^	2.169 (1.102,4.269)^*^
27.10~<27.78	2.880 (1.555,5.333)^**^	2.679 (1.384,5.185)^**^	2.372 (1.232,4.568)^*^
27.78~<28.89	4.377 (2.401,7.981)^***^	4.236 (2.271,7.901)^***^	3.842 (2.056,7.178)^***^
28.89~<30.45	4.101 (2.244,7.496)^***^	3.643 (1.904,6.969)^***^	3.306 (1.733,6.307)^***^
≥30.45	4.312 (2.371,7.843)^***^	4.061 (2.177,7.577)^***^	3.897 (2.085,7.281)^***^

^a^Crude HR; ^b^Sex adjusted; ^c^Sex and age adjusted; ^*^ *P* < 0.05; ^**^ *P* < 0.01; ^***^ *P* < 0.001.

**Table 4:** Hazard ratio for T2DM incidence of BMI in aminotransferase group 2

	Hazard Ratio (95% CI)		
	Model 1^a^	Model 2^b^	Model 3^c^
**BMI *kg/m^2^***			
<19.05	0.591 (0.306,1.143)	0.599 (0.310,1.158)	0.653 (0.337,1.263)
19.05~<19.96	0.523 (0.271,1.012)	0.538 (0.278,1.041)	0.564 (0.291,1.091)
19.96~<20.66	1	1	1
20.66~<21.22	0.931 (0.525,1.651)	0.908 (0.512,1.610)	0.884 (0.498,1.567)
21.22~<21.72	1.032 (0.595,1.788)	0.998 (0.575,1.730)	0.954 (0.550,1.653)
21.72~<22.22	1.255 (0.724,2.175)	1.169 (0.674,2.029)	0.994 (0.571,1.732)
22.22~<22.67	1.188 (0.689,2.050)	1.095 (0.633,1.891)	0.928 (0.535,1.612)
22.67~<23.14	1.184 (0.672,2.085)	0.955 (0.537,1.700)	0.894 (0.503,1.590)
23.14~<23.56	1.304 (0.756,2.249)	1.115 (0.641,1.940)	0.948 (0.543,1.654)
23.56~<24.03	1.362 (0.782,2.373)	1.062 (0.597,1.891)	0.887 (0.498,1.581)
24.03~<24.46	1.898 (1.138,3.165)^*^	1.469 (0.862,2.504)	1.244 (0.727,2.129)
24.46~<24.91	1.926 (1.141,3.248)^*^	1.409 (0.813,2.444)	1.214 (0.699,2.111)
24.91~<25.39	2.530 (1.530,4.184)^***^	1.912 (1.116,3.277)^*^	1.366 (0.800,2.333)
25.39~<25.91	3.015 (1.848,4.920)^***^	2.345 (1.397,3.935)^**^	1.874 (1.113,3.154)^*^
25.92~<26.45	3.030 (1.848,4.968)^***^	2.217 (1.286,3.823)^**^	1.669 (0.966,2.883)
26.45~<27.10	3.307 (2.010,5.443)^***^	2.737 (1.584,4.728)^***^	2.212 (1.278,3.829)^**^
27.10~<27.78	2.694 (1.609,4.510)^***^	1.855 (1.056,3.258)^*^	1.514 (0.863,2.657)
27.78~<28.89	3.460 (2.119,5.648)^***^	2.913 (1.713,4.954)^***^	2.160 (1.271,3.669)^**^
28.89~<30.45	4.508 (2.780,7.309)^***^	3.506 (2.074,5.927)^***^	2.810 (1.656,4.767)^***^
≥30.45	6.903 (4.327,11.011)^***^	6.248 (3.805,10.257)^***^	5.142 (3.096,8.539)^***^

^a^Crude HR; ^b^Sex adjusted; ^c^Sex and age adjusted; ^*^ *P* < 0.05; ^**^ *P* < 0.01; ^***^ *P* < 0.001.

**Figure 1:**
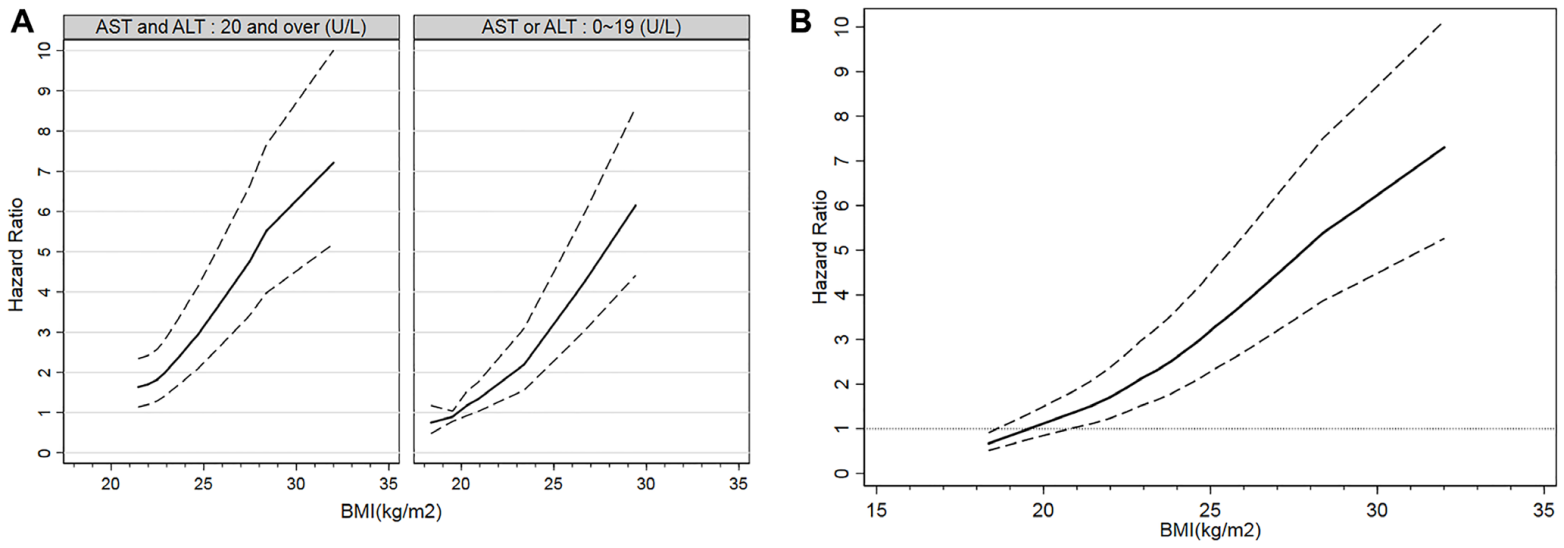
Incidence of T2DM according to BMI. Hazard ratios and 95% CIs of T2DM incidence according to BMI were shown. (**A)** Incidence of T2DM according to BMI by aminotransferase groups. (**B**) Incidence of T2DM according to BMI in all participants.

The HR of T2DM according to BMI was shown in Figure 1. Figure 1A showed the risk of T2DM incidence according to BMI in aminotransferase group 1 and group 2; The slop of curve increased when BMI exceeded 23.4 kg/m^2^ in group 2; while the slop of curve decreased when BMI exceeded 28.4 kg/m^2^. Figure 1B showed the that in the total participants; The slop increased when BMI exceeded 22.0 kg/m^2^ and then decreased when BMI exceeded 28.5 kg/m^2^. The turning point of BMI at high risk of T2DM was 25.0 (HR 2.699; 95% CI 1.072~6.642) kg/m^2^. In aminotransferase group 2, the turning point of BMI was 25.1 (HR 3.213; 95% CI 1.140~9.050) kg/m^2^, and that in group 1 was 26.1 (HR 4.935; 95% CI 2.041~11.930) kg/m^2^. These findings suggested that the relationship between BMI and T2DM incidence was greater in those with AST < 20 or ALT < 20.

## DISCUSSION

BMI control is an issue of concern in T2DM prevention, multiple observational studies has reported the association between BMI and T2DM [7–11, 14, 17, 25]. Consist with the findings in these studies, higher BMI was associated with increased incidence of T2DM in our study. The cumulative incidence of T2DM was less than 2% for BMI < 20.66 kg/m^2^ and was more than 5% for BMI ≥ 25.39 kg/m^2^. HR also increased with BMI, and was higher than 2 for BMI over 24.03 kg/m^2^. Guidelines for the prevention and control of type 2 diabetes in China (2017 Edition) points out that 25.0 kg/m^2^ was the average BMI for T2DM patients, while the average baseline BMI for T2DM development in our study was 26.22 kg/m^2^. The probable cause of the difference might be the multiple nationalities in Xinjiang and the geographical location of Central Asia. The Chennai Urban Rural Epidemiology Study suggested a BMI cut-off value of 24.0 associated with increased risk of hypertension, dyslipidemia and diabetes in Chinese adults [26]. In our study, the BMI cut-off value was 25.0 kg/m^2^, 1.0 kg/m^2^ higher than the suggested 24.0 kg/m^2^, indicating a weakened risk of T2DM for the same BMI value in Xinjiang population. Our finding suggested that in Xinjiang population, 25.0 kg/m^2^ may be an key BMI value in T2DM development. It also suggested that people with BMI over 25 kg/m^2^ had higher predisposition to T2DM and especially need intervention to loss weight.

Liver plays a major role in the regulation of glucose homeostasis by regulating various pathways of glucose metabolism, including gluconeogenesis, glycogenolysis, glycogenesis and glycolysis [27, 28]. Studies have proved that among the most studied clinical liver serum bio-markers(AST, ALT, gamma-glutamyl transpeptidase, alkaline phosphatase), higher serum AST or ALT is associated with higher T2DM risk [15–17]. In our study, we set 19 U/L as healthy limit for participants and set aminotransferase group 1 as higher level for both AST and ALT. In our study, the rise of both AST and ALT (group 1) was an independent risk factor of T2DM. Since serum AST and ALT are markers of non-alcoholic fatty liver disease (NAFLD), which is a strong independent risk factor of T2DM, this finding can be explained. Elevated serum AST and ALT alone could also increase the risk of T2DM, and is independent of NAFLD [29]. BMI in group 2 showed higher association (higher HR) with T2DM compared with group 1. Also, the cut-off point of BMI in group 1 (26.1 kg/m^2^) is higher than that in group 2 (25.1 kg/m^2^), indicating a greater relationship of BMI and T2DM in group 2, probably because of insulin resistance caused by liver dysfunction [28].

## MATERIALS AND METHODS

### Study population

We conducted a retrospective cohort study based on check-up patients in Karamay Central Hospital of Xinjiang from 2008 to 2017. Baseline age, sex, fasting blood sugar (FBS), height, weight, AST, ALT and annual FBS were collected. Patients with baseline FBS ≥ 7.0 mmol/L were excluded and 33346 participants with FBS < 7.0 mmol/L were included in the study. Our study was approved by the Ethics Committee of the Karamay Center Hospital of Xinjiang.

### Statistical analysis

Baseline BMI were calculated by baseline height and weight, T2DM was diagnosed as FBS ≥ 7.0 mmol/L. Sex group were set as male and female. Age group were set as under 30, 30~44, 45~59 and 60 years old or over. Baseline BMI were divided into 20 groups by percentiles. We divided AST and ALT by 20 U/L. According to AST and ALT, we set aminotransferase group 1 and group 2. All analyses were conducted separately for totality, group 1 and group 2. We analyzed the incidence of T2DM in each sex group, age group, BMI group and aminotransferase group. We also estimated the crude hazard ratio (HR), sex-adjusted HR and age-and-sex-adjusted HR of T2DM for each BMI group. We set BMI categories by 5th 10th 15th 20th 25th 30th 35th 40th 45th 50th 55th 60th 65th 70th 75th 80th 85th 90th 95th 100th percentiles to estimate the non-linear association between BMI and T2DM for totality, group 1 and group 2. We determine the turning point of BMI at high risk of T2DM as the lowest BMI value for which the HR of T2DM was statistically significant with a reference BMI of 20.00 (19.95~<20.05) kg/m^2^ and the increment was 0.1 kg/m^2^.

All statistical analysis were conducted by STATA 15.0 and IBM SPSS Statistics 26.0.

## Abbreviations

T2DM: type 2 diabetes
BMI: body mass index
FBS: fasting blood sugar
AST: aspartate aminotransferase
ALT: alanine aminotransferase
HR: hazard ratio
SD: standard deviation
